# TECHNOLOGY AND PROSPECTIVE MEMORY: ATTITUDES AMONG OLDER ADULTS WITH SUBJECTIVE COGNITIVE IMPAIRMENT

**DOI:** 10.1093/geroni/igae098.3731

**Published:** 2024-12-31

**Authors:** Edie Sanders, Walter Boot

**Affiliations:** Weill Cornell Medicine, New York, New York, United States; Weill Cornell Medicine, New York, New York, United States

## Abstract

Prospective memory (PM), the ability to remember to execute an intention in the future, is critical for the performance of everyday tasks important for independence and quality of life. PM failures are more frequent with increased age and can be even greater for older adults with a cognitive impairment such as subjective cognitive decline (SCD). Technology holds promise for providing everyday PM support for older adults with SCD, but little is known regarding their attitudes and preferences toward technology-based PM support. The present work utilized an online survey and focus groups to examine these attitudes. 188 older adults with SCD and 190 older adults without SCD completed an online survey on a variety of technologies to support PM in different areas of activities. A separate sample of 24 older adults with SCD participated in online focus groups and were probed about their perception of the use of technology devices for supporting PM. Results show that the attitudes of older adults with SCD 1) differed based on the type of device and the type of activity the technology is meant to support, 2) were more positive than attitudes of older adults without SCD, and 3) were associated with a variety of individual difference factors. Results indicate that older adults with SCD may be a promising population to target using technology-based solutions, and the type of device and type of activity being supported are important to consider in the development of technology solutions to enhance independence and well-being.

